# Novel PVA–Hyaluronan–Siloxane Hybrid Nanofiber Mats for Bone Tissue Engineering

**DOI:** 10.3390/polym16040497

**Published:** 2024-02-11

**Authors:** Daniela Anahí Sánchez-Téllez, Shantal Lizbeth Baltierra-Uribe, Mónica Araceli Vidales-Hurtado, Alejandra Valdivia-Flores, Blanca Estela García-Pérez, Lucía Téllez-Jurado

**Affiliations:** 1 Department of Engineering in Metalurgy and Materials, Escuela Superior de Ingeniería Química e Industrias Extractivas, Instituto Politécnico Nacional, Unidad Profesional Adolfo López Mateos (UPALM), Av. Instituto Politécnico Nacional S/N, Zacatenco, Mexico City 07738, Mexico; 2 Department of Microbiology, Escuela Nacional de Ciencias Biológicas, Instituto Politécnico Nacional, Prolongación de Carpio y Plan de Ayala S/N, Casco de Santo Tomás, Mexico City 11340, Mexico; 3 Centro de Investigación en Ciencia Aplicada y Tecnología Avanzada, Unidad Querétaro, Instituto Politécnico Nacional, Cerro Blanco 141, Colinas del Cimatario, Santiago de Querétaro 76090, Mexico

**Keywords:** nanofibers, electrospinning, hyaluronic acid, polyvinyl alcohol, siloxanes, hybrids, osteoblasts

## Abstract

Hyaluronan (HA) is a natural biodegradable biopolymer; its biological functions include cell adhesion, cell proliferation, and differentiation as well as decreasing inflammation, angiogenesis, and regeneration of damaged tissue. This makes it a suitable candidate for fabricating nanomaterials with potential use in tissue engineering. However, HA nanofiber production is restricted due to the high viscosity, low evaporation rate, and high surface tension of HA solutions. Here, hybrids in the form of continuous and randomly aligned polyvinyl alcohol (PVA)–(HA)–siloxane nanofibers were obtained using an electrospinning process. PVA–HA fibers were crosslinked by a 3D siloxane organic–inorganic matrix via sol-gel that restricts natural hydrophilicity and stiffens the structure. The hybrid nanofiber mats were characterized by FT-IR, micro-Raman spectroscopy, SEM, and biological properties. The PVA/HA ratio influenced the morphology of the hybrid nanofibers. Nanofibers with high PVA content (10PVA-8 and 10PVA-10) form mats with few beaded nanofibers, while those with high HA content (5PVA-8 and 5PVA-10) exhibit mats with mound patterns formed by “ribbon-like” nanofibers. The hybrid nanofibers were used as mats to support osteoblast growth, and they showed outstanding biological properties supporting cell adhesion, cell proliferation, and cell differentiation. Importantly, the 5PVA-8 mats show 3D spherical osteoblast morphology; this suggests the formation of tissue growth. These novel HA-based nanomaterials represent a relevant advance in designing nanofibers with unique properties for potential tissue regeneration.

## 1. Introduction

Hyaluronic acid (HA) is an important component of the extracellular matrix (ECM) in the human body [1]. Some properties of HA such as viscoelasticity, high moisture absorption capability and hygroscopic nature, make HA an excellent biopolymer for maintaining the viscoelasticity of the ECM, supporting cellular structure, and functioning as a lubricant [2]. Recently, HA has gained attention in the design of novel devices for application in the bioengineering and medical fields due to its exceptional physicochemical and biological attributes [3]. HA has been widely used in bone regeneration therapies, serving as either hyaluronic acid-incorporated scaffolds [3] or as carriers for cell seeding and/or bioactive components [4]. Even though hyaluronic acid itself has a limited effect on osteogenesis enhancement, HA derivatives and HA-incorporated hybrid scaffolds have shown the potential to improve osteogenesis and mineralization [5]. Based on current research, it is observed that HA is becoming the key biopolymer component in HA-based hybrid scaffolds [6,7] as it improves their biological osteoinductive properties.

The design of scaffolds from biocompatible nanofibers represents an excellent advantage, because they have a porous and 3D structure, and the high surface-to-volume ratio creates a more suitable microenvironment for cell growth [8]. Several in vitro studies have shown that these structures are ideal for cell adhesion, proliferation, and migration [9,10,11,12]. Moreover, this type of 3D microenvironment is necessary for nutrient transport, cell ingrowth, and vascularization [13,14].

Along with the composition of the scaffolds, the process of manufacturing fiber materials is important. One of the techniques used to produce micro/nanofiber mats in a continuous process is the electrospinning technique. This process allows for the design of fibers with diameters adjusted from nanometers to microns. The fiber morphologies and sizes can be regulated by tuning the following parameters: (1) solution parameters including viscosity, solvent, polymer concentration, molecular weight of the polymer, conductivity, and surface tension; (2) processing parameters including applied voltage, distance between tip and collector, needle diameter, feed rate/flow rate, and collector type; (3) ambient parameters including humidity and temperature [15,16].

Unfortunately, electrospinning of nanofibrous mats made up purely of biopolymers is difficult to achieve because very low polymer concentrations reach high solution viscosities [17]. However, to take advantage of the HA viscoelastic and biological properties, HA can be chemically modified and/or physically blended with other polymer solutions (PVA, PCL, PLA) to make it electrospinnable. Moreover, the need to use organic solvents rather than water or alcohol to obtain the critical chain entanglement concentration necessary to being electrospun reduces the nanofibers’ biological and biomedical applicability. Sun et al. [18] successfully electrospun nanofibers from an aqueous complex coacervate solution composed of chitosan and hyaluronic acid. They eliminated the need for toxic solvent systems in the electrospun biopolymer solution and the risk of degradation effects.

Due to the excellent biocompatibility of polyvinyl alcohol (PVA), PVA-based hydrogels have been tried out as tissue regeneration materials such as artificial articular cartilage, intervertebral discs, and meniscus [19]. In addition, PVA has been shown to form easily electrospun fibers [19]. However, like other natural and synthetic polymers, its degradation rates can hardly be designed or adjusted to match the regeneration rates of bone tissue. Thus, to improve degradation resistance, mechanical properties, and osteoconductivity, polymers can be hybridized with inorganic components (hydroxyapatite [20], biomimetic calcium phosphate, ${SiO}_{2}$ [21]) or crosslinked with silicone (PDMS)-modified silica (${SiO}_{2}$) 3D networks. Organic–inorganic materials are classified as hybrids or nanocomposites because organic and inorganic components are bonded in a single system at the molecular or nano level. Under optimal conditions, hybrid materials show the synergy of the properties of both components. Although this type of crosslinking has produced excellent results, the hybridization of biopolymers (sodium hyaluronate, chondroitin sulfate, chitosan, alginate) [22,23] or synthetic polymers (PVA, PLA, PCL, PEG) is rarely explored. Sánchez-Téllez et al. [6] proved in their study that the crosslinking of polysaccharide chains by a 3D siloxane organic–inorganic matrix via the sol-gel method gave sodium hyaluronate matrix strength, improving rheological properties and increasing thermal degradation temperatures. 

Because the degradative and biological properties of hybrids can be modulated, the properties of electrospun nanofiber mats built up from an organic (sodium hyaluronate, PVA)–inorganic (siloxane) hybrid solution are magnified due to their high surface areas that enable the deposition of cell adhesive molecules (peptides) on the surface. This increases cell adhesion, promotes cell proliferation, and maintains cell phenotype. Therefore, to increase the desirable physicochemical and biological properties of hybrid materials, the aim of this work was to design 3D cell scaffolds based on hybrid electrospun nanofiber mats composed of PVA–HA hydrogels inorganically crosslinked by a PDMS-modified SiO_2_ organic–inorganic network. The fabricated mats showed that they promote cell adhesion, proliferation, and mineralization of human osteoblasts; this highlights their potential use in bone tissue engineering. 

## 2. Materials and Methods

Techniques and equipment used for the processing, characterization, and determination of chemical and biological properties of the hybrid hydrogels are described.

### 2.1. Materials 

Electrospun PVA–HA hybrid nanofiber mats were prepared by chemical modification and hybridization of PVA–HA and then inorganically crosslinked via the sol-gel method. The starting reagents were: (1) sodium hyaluronate oral grade with a Mw = 150–300 kDa, PDI = 1.3 [24] (HA, Sigma-Aldrich, St. Louis, MO, USA); (2) N-(3-Dimethylaminopropil)-N’ethylcarbodiimide hydrochloride (EDC, C_8_H_17_N_3_∙HCl, Sigma-Aldrich); (3) N-hydroxysuccinimide (NHS, C_4_H_5_NO_3_, Sigma-Aldrich); (4) 2-(N-Morpholino) ethanesulfonic acid hydrate (MES, C_6_H_13_NO_4_S∙H_2_O, Sigma-Aldrich); (5) 3-aminopropyltriethoxysilane (APTES, H_2_N(CH_2_)_3_Si(OCH_2_CH_3_)_3_, Sigma-Aldrich); (6) deionized water; (7) hydrolyzed polyvinyl alcohol (PVA, Mw = 77,000–79,000 Da, J.T. Baker, Mallinckrodt Baker, Inc., Phillipsburg, NJ, USA), PDI = 2–2.5 [25]; (8) tetraethylorthosilicate (TEOS, Si(OCH_2_CH_3_)_4_, Sigma-Aldrich); (9) hydroxyl-terminated polydimethylsiloxane (PDMS, OH-[Si(CH_3_)_2_-O]nH, Mw = 550 gmol, viscosity = 25 cSk, Sigma-Aldrich), PDI = 1.17 [26]. 

### 2.2. Synthesis of the PVA–HA–Siloxane Hybrid Sol

For the synthesis process of PVA–HA hybrid nanofiber mats, two starting solutions were needed. For the first solution, an adequate amount of PVA (7% *w*/*v*) is dissolved into deionized water at 80 °C under constant stirring. The PVA aqueous solution was allowed to cool down until it reached room temperature. For the second solution [6], an adequate amount of HA (3% *w*/*v*) was dissolved into deionized water at room temperature. Then, a chemical modification of the HA was made by the amidation of its carboxyl groups (R-COO-Na) with the amine groups of the inorganic modifier APTES. Thus, EDC and NHS were added to the HA aqueous solution that acts as a carboxylic acid activating agent. After 30 min, the accurate amount of MES and APTES were also added to the solution [27]. Molar ratios of the HA/APTES/EDC/NHS/MES used are 1/1/1.5/1/2.5, respectively. Inorganic crosslinking of HA occurred using the sol-gel method by adding TEOS and PDMS to the solution; this led to a hybrid solution with a 3D siloxane organic–inorganic matrix being obtained. The weight ratios of the components APTES/TEOS/PDMS used are 0.1/0.1/0.8, respectively. Finally, both solutions were mixed using the following relations: PVA:HA = 10:1 and 5:1. The hydrolysis–condensation (crosslinking) reactions took place among PVA, TEOS, APTES, and PDMS [28,29,30,31] at room temperature under stirring conditions for 1 h. After that, the solution was dialyzed (MWCO of 6000–8000, Spectrum^®^ Laboratories, Piscataway, NJ, USA) using ultrapure water to eliminate the excess of EDC/NHS. The resulting sol was poured into a plastic syringe for electrospinning injection.

### 2.3. Preparation of the Electrospun PVA–HA–Siloxane Nanofiber Mats

The prepared sols were electrospun to form organic–inorganic crosslinked nanofibers. The electrospinning equipment (Basic Electrospinning Series) consists of a high-voltage power supply, a syringe infusion pump, and a ground electrode (aluminum foil on a stainless-steel collector). The sols were loaded into a plastic syringe (10 mL). The experimental conditions such as needle–collector distance, electric field strength, and flow rate were optimized to achieve the best nanofiber formation. The voltage used was 7.5 kV. The solution was constantly supplied at a flow rate of 2.5 mL/h with a needle (27 G) 0.4 mm × 12 mm. The spinning distance between the needle tip and the ground electrode was 8 and 10 cm. Under these conditions, the obtained fiber network is 0.1–0.5 μm thick. The sample code of each hybrid mat is assigned according to (1) PVA–HA ratio and (2) spinning distance, e.g., sample 10PVA-8 that indicates a ratio of PVA:HA = 10:1 and obtained at a spinning distance of 8 cm.

### 2.4. Characterization of PVA–HA–Siloxane Nanofiber Mats

Structural characterization of PVA–HA hybrid nanofiber mats was performed by FT-IR, SEM, and micro-Raman spectroscopy. FT-IR spectra were obtained at room temperature in a Perkin Elmer Spectrum Two spectrometer coupled with an Attenuated Total Reflection, Perkin Elmer (Hong Kong) (ATR-FTIR). Spectra were recorded in transmittance mode in the range from 4000 to 600 cm^−1^ by 16 scans and with a resolution of 4 cm^−1^. Morphological characterization of PVA–HA hybrid mats was performed in a high-resolution JEOL Scanning Electron Microscope, (JEOL, Peabody, MA, USA). The Raman spectra were obtained in a micro-Raman confocal LabRam (Horiba Jobin Yvon, Oberursel, Germany) coupled with microscopy.

### 2.5. Cell Culture

Human osteoblast cell line MG-63 (CRL-1427) was obtained from the American Type Culture Collection (ATCC, Rockville, MD, USA). Osteoblasts were cultured in α-Minimum Essential Medium Eagle (α-MEM, Sigma Aldrich, St. Louis, MO, USA) supplemented with 10% heat-inactivated fetal bovine serum (FBS, Gibco, Thermo Fisher Scientific, Waltham, MA, USA) under a 5% CO_2_ atmosphere at 37 °C. Media was renewed each third day. When cells reached confluence, they were detached from culture flasks using 0.05 g/L trypsin and 0.05 g/L EDTA solution (Sigma Aldrich).

#### 2.5.1. Cell Culture on PVA–HA–Siloxane Nanofiber Mats

Mat samples of 0.5 cm^2^ were sterilized first by immersion into a 70% ethylic alcohol solution for 15 min and then by exposure under ultraviolet light for 30 min. Then mats were incubated with FBS-supplemented α-MEM media for 2 days. Media was changed each day. MG-63 osteoblast cells were cultured on the mats at a density of 5 × 10^4^ cells per sample under a 5% CO_2_ atmosphere at 37 °C. The evaluation of the cell viability, cytoskeletal arrangement, and cell attachment was performed at 24 h, 7, 14, and 21 days of culture. The media was changed every day until the experiment finished. Three replicates of each sample were tested.

#### 2.5.2. Cell Viability on PVA–HA–Siloxane Nanofiber Mats

MG-63 osteoblast cells grown onto the nanofiber mats for 1, 7, 14, and 21 days were labeled with a mix of nucleic acid dyes (SYTO9; 5 μmol/L; Invitrogen, Carlsbad, CA, USA) and propidium iodide (PI; 30 μmol/L; Sigma Aldrich) to allow visualization of living and dead cells [32]. The samples were observed with a confocal laser scanning microscopy system (LSM5 Pascal, Zeiss, Jena, Germany). 

#### 2.5.3. Stain of Actin Filaments in Cells Adhered onto PVA–HA–Siloxane Nanofiber Mats

MG-63 osteoblast cells grown onto the nanofiber mats for 1, 7, 14, and 21 days were fixed with 4% paraformaldehyde solution (Sigma Aldrich) and then washed three times with PBS. Actin filaments were stained with phalloidin-tetramethylrhodamine B isothiocyanate (phalloidin-TRITC; 10 ng/μL; Sigma Aldrich) for 20 min at room temperature. The excess of the phalloidin-TRITC was eliminated by washing the nanofiber mats five times with PBS. Nuclei of cells were counterstained with DAPI (Vector Laboratories, Burlingame, CA, USA). The samples were observed with a confocal laser scanning microscopy system (LSM5 Pascal, Zeiss, Germany).

#### 2.5.4. Scanning Electron Microscopy of Cells Adhered on PVA–HA Hybrid Nanofiber Mats

MG-63 osteoblast cells were grown onto the nanofiber mats for 1, 7, 14, and 21 days and were fixed with 2.5% glutaraldehyde solution and then post-fixed with 1% aqueous osmium tetroxide solution. Samples were washed three times and then dehydrated with increasing concentrations of ethyl alcohol solutions and critical point drying with liquid CO_2_. Finally, for observation through scanning electron microscopy (SEM, 5800LV GenTech Scientific, Arcade, NY, USA), preparations were coated with gold in a sputter coater (Denton Vacuum-Desk II, Moorestown, NJ, USA).

#### 2.5.5. Calcium Deposits on PVA–HA Hybrid Nanofiber Mats

MG-63 osteoblast cells were grown onto the nanofiber mats for 1, 7, 14, and 21 days and were fixed with 4% paraformaldehyde solution for 15 min at room temperature. Then samples were washed with PBS solution to eliminate the excess of paraformaldehyde. The mineralization process was evidenced by the alizarin assay; the is considered the gold standard for quantification of osteoblast mineralization [33]. In brief, 200 μL of 40 mM alizarin red was added to each sample and incubated for 20 min at room temperature. Samples were washed 5 times with PBS to remove the colorant. Finally, samples were analyzed under an inverted microscope (Primovert, Zeiss, Jena, Germany).

## 3. Results and Discussion

### 3.1. Synthesis of the PVA–HA–Siloxane Hybrid Precursor Sol

The hybrid sol was prepared by the hybridization and crosslinking of PVA-modified HA polymers with organic–inorganic siloxane compounds: 3-aminopropyltriethoxysilane (APTES), tetraethylorthosilicate (TEOS), and polydimethylsiloxane (PDMS). The preparation worked as follows: (1) the amidation of sodium hyaluronate (HA) with the inorganic modifier APTES to form amide functional groups: HA-APTES modified molecules; (2) the hydrolysis of siloxane organic–inorganic precursors: HA-APTES modified molecules–silica alkoxide [TEOS]–silicone [PDMS] [6]; (3) the condensation of previously hydrolyzed organic–inorganic precursors among them and/or with PVA to form the 3D PVA-modified HA siloxane organic–inorganic hybrid sol [34,35,36]. 

**Figure 1:**
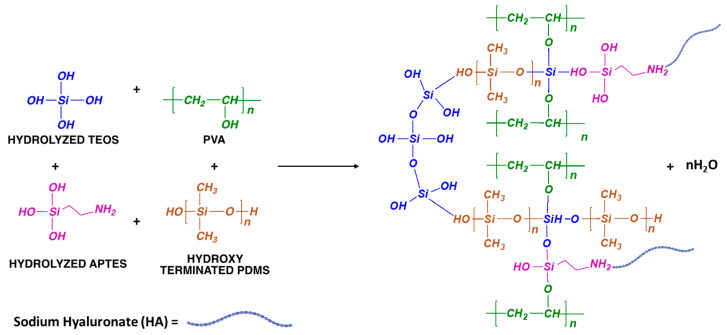
Scheme of the possible PVA-modified HA polymeric matrix crosslinked with a siloxane organic–inorganic 3D network.

The ethoxy groups $({-OC}_{2}H_{5}$) from APTES and TEOS are hydrolyzed by water molecules to form silanol groups $\left(Si-OH\right)$. Simultaneously, an ethanol molecule $\left(C_{2}H_{5}-OH\right)$ is formed per each hydrolyzed radical. Then, silanol groups $\left(Si-OH\right)$ either from hydrolyzed APTES-TEOS molecules or hydroxyl-terminated PDMS chains condense among them or with -OH groups from PVA; this results in a hybrid sol (Figure 1) that will become the precursor of a PVA–HA polymeric matrix crosslinked with a siloxane organic–inorganic 3D network. Because the rate of hydrolysis reactions is faster than the reaction rate of condensation reactions, the condensation process is finished during electrospinning when the nanofibers are formed.

### 3.2. Characterization of the PVA–HA–Siloxane Hybrid Nanofiber Mats

**Figure 2:**
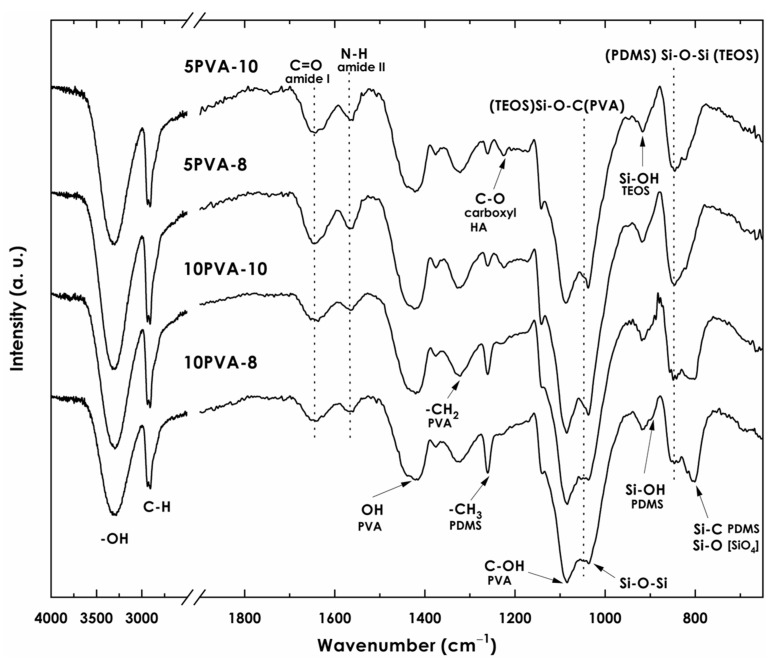
FT-IR spectra of electrospun PVA–HA–siloxane hybrid nanofibers. Nanofibers were obtained from relation 10:1 and 5:1 PVA:HA and spinning distances of 10 cm (10PVA-10) and 8 cm (10PVA-8). The principal bands associated with obtaining the PVA–HA–siloxane hybrid material are shown.

Hybrid nanofiber mats were obtained by electrospinning of a PVA–HA–siloxane solution. The electrospinning process allowed fibers to be shaped by the coaxial stretching of the PVA–HA–siloxane colloidal solution, in which sol-gel reactions took place to form the 3D hybrid organic–inorganic network. Bands related to the intermolecular interactions of PVA, sodium hyaluronate, APTES, and siloxane network formed during hydrolysis-condensation reactions are observed in the infrared spectra (Figure 2) of the hybrid nanofiber membranes. The assignation of bands was performed according to the literature data. Bands related to PVA are observed: (1) the wide band with a maximum at 3320 cm^−1^ due to stretching vibrations $\left(\vartheta_{O-H}\right)$ of –OH bonds from intermolecular and intramolecular hydrogen bonds; (2) the band at 2920 cm^−1^ attributed to asymmetric stretching ${(\vartheta }_{a\;C-H})$ of C−H bonds from alkyl groups; (3) the band at 1430 cm^−1^ corresponding to the deformation vibration of –OH; (4) the band at 1315 cm^−1^ related to the scissoring vibration of ${-CH}_{2}$ in PVA backbone; (5) the band at 1084 cm^−1^ due to the stretching vibrations ${(\vartheta }_{C-OH})\;$of alcohol in PVA; (6) the band at 836 cm^−1^ attributed to the out-of-plane vibration of −CH bonds [21]. Other relevant bands demonstrating the chemical bonding between macromolecules of PVA and HA polymers and the siloxane organic–inorganic network to form the nanofiber mats can be observed in the FT-IR spectra: (1) the band at 1560 cm^−1^ is assigned to bending vibrations $\left(\delta_{N-H}\right)$ of amide II due to N−H groups [37,38]. This results from the chemical modification of sodium hyaluronate with APTES [6,27] and confirms the presence of HA-APTES bonding within the nanofiber chemical composition; (2) from hydrolysis reactions, the bands at 920 cm^−1^ and 895 cm^−1^ are assigned to Si−OH stretching due to silanol groups in hydrolyzed TEOS and to asymmetric stretching $\vartheta_{a}\left(Si-O\right)$ in Si−OH bonds from hydroxyl-terminated PDMS, respectively; (3) the formation of siloxane structures (Q-D units) due to condensation is observed at 850 cm^−1^, corresponding to (TEOS) Si−O−Si (PDMS) bonds; (4) the band at 1100 cm^−1^ can be assigned to both asymmetric stretching $\left(\vartheta_{a\;Si-O}\right)$ in Si−O−Si bonds from siloxanes and asymmetric stretching $\left(\vartheta_{a\;Si-O-C}\right)$ in Si−O−C due to condensation of PVA with siloxane molecules [21]; (5) the band at 790 cm^−1^ assigned to Si−O bonds due to silicon tetrahedron $\left[{SiO}_{4}\right]$ and Si−C bonds due to PDMS and APTES molecules [6,28,30].

The composition of each PVA–HA–siloxane hybrid sol precursor influenced the chemical bonding between the polymer macromolecules (PVA, HA) and the siloxane (PDMS-TEOS) organic–inorganic hybrid matrix in the nanofiber mat. Condensation between different polymers competes with the condensation between molecules of the same polymer and/or the condensation with silanol groups from hydrolyzed TEOS and the hydroxyl-terminated PDMS. 

Nanofiber mats with lower HA content and, therefore, more PVA content, (10PVA-8, 10PVA-10) showed more intense bands related to PDMS (1259, 895, 790 cm^−1^) in comparison to mats with more HA content (5PVA-8, 5PVA-10). The band at 895 cm^−1^ attributed to Si−OH from hydroxyl-terminated PDMS is more intense in mats with higher PVA content, meaning that a larger amount of PDMS chains did not condense with −OH groups from TEOS and/or APTES in the presence of PVA. This suggests a higher affinity of PVA with PDMS. That may be confirmed by the band at 790 cm^−1^ showing freer [SiO_4_] tetrahedra. However, in mats with lower PVA content, as the band at 895 cm^−1^ decreased in intensity, the band at 850 cm^−1^ attributed to siloxane D-Q units increased, showing higher (PDMS) Si−O−Si (TEOS) siloxane bonding, and thus a more condensed network. Moreover, bands related to HA (1640, 1560, 1220 cm^−1^) appear to be more intense in mats with more HA content, indicating that more HA chains reacted with the hybrid siloxane system. Since the bands at 1640 cm^−1^ and 1220 cm^−1^ attributed to C=O and COO^−^ groups from HA, respectively, show higher intensity in comparison to the band at 1560 cm^−1^ attributed to amide II, HA chains were partially modified and hybridized by APTES. Thus, unhybridized HA macromolecules could have formed two types of hydrogen bonds with PVA polymer macromolecules [39]; that is, between two hydroxyl groups (PVA) OH−OH (HA) and/or between the amide groups and the oxygen of the hydroxyl groups (HA) NH−OH (PVA). Therefore, the broadening or shifting of characteristic bands attributed to HA and PVA may be related to the interaction between them [21]. Finally, unreacted polymeric chains from PVA, HA, and PDMS may coexist in equilibrium by electrostatic forces.

**Figure 3:**
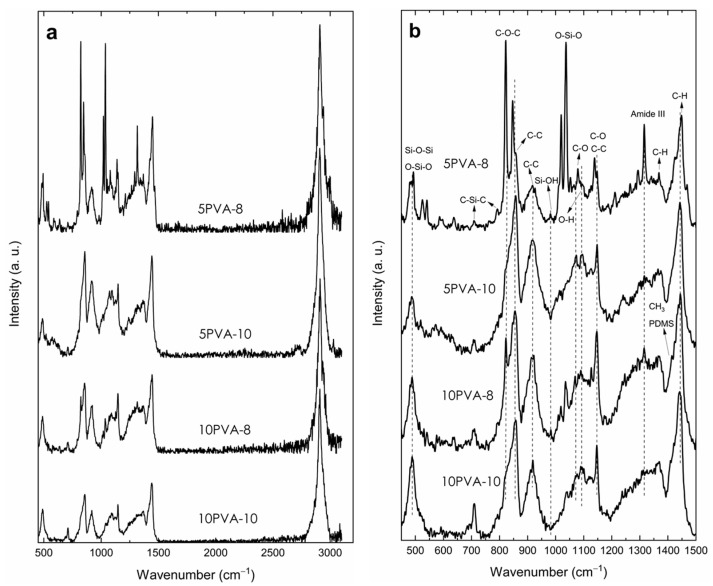
Micro-Raman spectra of electrospun PVA–HA–siloxane hybrid nanofibers. Hybrid nanofibers were obtained from relations 10:1 and 5:1 PVA:HA at spinning distances of 10 (10PVA-10 and 5PVA-10) and 8 cm (10PVA-8 and 5PVA-8). (**a**) 450–3200 cm^−1^ range. (**b**) Region below 1500 cm^−1^. The sharper and more intense bands of the sample 5PVA-8 denote a higher structural order.

Figure 3a shows Raman spectra of electrospun PVA–HA–siloxane hybrid nanofibers in the range of 450 to 3200 cm^−1^. In this region, there is a set of bands that, like the results shown in IR, present evidence of the transformation process of the precursors present in the chemical mixture for the formation of an organic–inorganic hybrid network. In general, it can be observed that the bands in the spectrum of sample 5PVA-8 are sharper and more intense, and this denotes a higher structural order. The band centered at 3000 cm^−1^ is the contribution of the vibrational modes of CH_2_ and CH_3_ from PVA and PDMS, respectively, and −OH groups of silanol (Si−OH) [40].

Details of the region where most of the bands are found, from 450 to 1500 cm^−1^, are shown in Figure 3b. The hydrolysis and condensation reactions of TEOS are evidenced by the two intense bands at 480 and 1037 cm^−1^ (the latter related to O−Si−O bonds), as well as two bands of very low intensity, one between 1122 and 1135 cm^−1^ (assigned to O−Si−O bonds bending) and the other centered on 980 cm^−1^ from Si−O stretching vibration of silanol groups (Si−OH). It is possible that the band in 1020 cm^−1^ can be associated with Si−O−C bond due to the integration of PVA into the network. The band at 480 cm^−1^ has been assigned to symmetric bending vibrations of the Si−O−Si linkage and/or to the O−Si−O deformation of the coupled ‘tetrahedra’ [SiO_4_] groups [41] due to condensation of hydrolyzed TEOS and PDMS. This band is generally intense and broad in SiO_2_ systems obtained by sol-gel; the full width at half maximum (FWHM) is associated with the wide distribution of Si−O−Si inter-tetrahedral angles within the structure. The decrease in its intensity indicates the lowering of network dimensionality by breaking Si−O−Si bridges within the glass structure [41]. In the spectra of all samples, this band is relatively narrow and of low intensity due to the interconnection with functional groups such as methyl and methylene of PDMS and PVA; these prevent the formation of the long-range Si−O−Si bridge network due to the formation of the hybrid compound. 

The low-intensity bands at 706 and 790 cm^−1^ are attributed to symmetric and asymmetric stretching vibrations, respectively, of C−Si−C bonds in PDMS [42]. The band at 821 cm^−1^ is related to stretching vibrations of C−O−C bonds from the hyaluronic acid repetitive units [6]. This structural part of the hybrid material is kept attached to the three-dimensional network through the APTES nitrogen atom by an amidation process, and this is confirmed by the amide III mode related to the band at 1314 cm^−1^ [43]. This band is sharper and more intense in samples 5PVA-8 and 10PVA-8, which is related to a higher structural order.

The bands related to PVA are those located at 856, 918, and 1147 cm^−1^; the first two are related to C−C stretching vibrations of the polymer backbone. In particular, the band at 918 cm^−1^ is associated with a non-crystalline conformation of PVA carbon backbone, so an increase in its intensity may represent a decrease in crystallinity. The band at 1147 cm^−1^ is assigned to C−C stretching vibration in the crystalline phase of the PVA matrix [17,44]. The intensity of the band at 918 cm^−1^ in the sample 5PVA-8 is lower than the rest of the samples, which confirms a higher structural order. Low-intensity bands are also seen at 1068 and 1092 cm^−1^ attributed to C−O stretching and O−H bending vibrations [45]. The low-intensity bands at 1365 cm^−1^ and the intense band at 1442 cm^−1^ are due to C−H and –OH bending vibrations from groups in PVA, PDMS, and hydrolyzed APTES [6,41,45].

**Figure 4:**
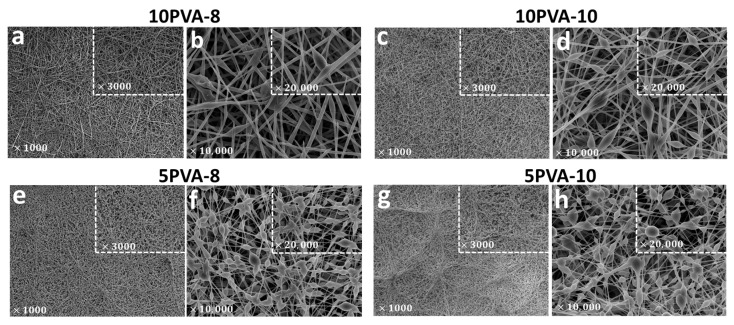
Ultrastructural morphology of electrospun PVA–HA–siloxane hybrid nanofiber mats. Images show the morphology difference between nanofibers. (**a**,**b**) Mats with hybrid nanofibers 10:1 PVA:HA and spinning distance of 8 cm. Mats show homogeneous nanofibers (**c**,**d**) Mats with hybrid nanofibers 10:1 PVA:HA and spinning distance of 10 cm. Mats show few beaded nanofibers. (**e**,**f**) Mats with hybrid nanofibers 5:1 PVA:HA and spinning distance of 8 cm. These mats show nanofibers beaded enrichment. (**g**,**h**) Mats with hybrid nanofibers 5:1 PVA:HA and spinning distance of 10 cm. These mats show a similar pattern to 5PVA-8 mats, with many beaded nanofibers. In all images, the square shows a magnification of the same sample. Magnification is indicated in each image.

The PVA–HA–siloxane hybrid nanofiber mats have nanometric organic–inorganic interpenetrating networks. Figure 4 displays SEM images, at different magnifications, of the morphology of the electrospun mats. Mats with lower HA content (10PVA-8 and 10PVA-10) exhibit homogeneous hybrid mats with few beaded nanofibers. In contrast, mats with more HA content (5PVA-8 and 5PVA-10) exhibit hybrid mats with mound patterns formed by beaded or “ribbon-like” nanofibers. Furthermore, the mats obtained with a shorter distance between the needle tip and the collector (10PVA-8 and 5PVA-8) resulted in nanofibers with thinner diameters. 

The formation of beadles nanofibers is attributed to (1) the increase of PVA concentration and the decrease of HA concentration (higher PVA concentration enhances the electrospinning behavior into the polymeric solution by shaping fibers with increased diameters [31,46]) within the polymeric blending gave the solution the adequate composition and thus a good polymer chain entanglement (at the established electrospinning parameters). It was, therefore, able to overcome surface tension and this resulted in the formation of almost uniform beadles electrospun nanofibers, as observed in 10PVA-8 and 10PVA-10 mats; (2) the stretching of the polymeric solution and the increase in distance between the needle tip and the collector both tailored smaller-diameter fibers, as observed in the 10PVA-10 mat compared to the 10PVA-8 mat. 

In contrast, the formation of beaded nanofibers (solvent–containing nanofibers) in mats with more HA content (5PVA-8, 5PVA-10) is attributed to the incomplete evaporation of the solvent (water/ethanol solution due to hydrolysis reactions in the sol-gel process) and the low-stretching of the polymeric blending in the flight between the needle and the metallic collector. Polymeric blending with a higher HA concentration (5PVA) appeared to be challenging to be electrospun [47] due to (1) the HA concentration within the polymeric blending increasing and changing miscibility and solution behavior [47,48]; (2) the decrease of PVA concentration allowed a higher concentration of colloidal particles within the polymeric solution when APTES–TEOS–PDMS started to be hydrolyzed and condensed during the sol-gel process. This resulted in faster gelation of PVA and HA polymers as viscosity increased in the sol, and this hindered electrospinning; (3) the decrease of PVA concentration might result in the reduction and breaking of entangled polymer chains before reaching the collector as the electric field is applied; (4) drying due to gelation of the polymeric solution at the tip hindering its flow through the needle tip and causing blockage; resulting in the formation of beaded nanofibers [15]. 

Bead morphology in all mats appear as elongated droplets or stretched droplets. This morphology is characteristic of viscous polymeric solutions [49]. However, slight changes in bead shapes are observed: in 10PVA-8 and 10PVA-10 mats, the few beaded nanofibers have stretched shapes, while in 5PVA-8 and 5PVA-10 mats, the abundant beaded nanofibers have more elongated shapes [15]. These variations are caused by concentration changes in the polymeric solution as PVA concentration decreases. Therefore, the concentration/viscosity of the polymeric solution determines the nanofibers’ morphology. Likewise, since the interactions between two macromolecules depend on the presence of a solvent [21], different evaporation rates of the solvent (water/ethanol) may lead to phase separation. This results in the fabrication of highly porous electrospun nanofibers, as observed in the four studied PVA–HA–siloxane hybrid nanofiber mats [15]. 

PVA-based nanofiber mats have limited applicability due to their high solubility in aqueous media. However, to take advantage of PVA’s electrospinning and biological properties [50], it is desirable to control and balance its hydrophilicity to modulate degradation rates and improved mechanical properties after being electrospun, shaping mats with structured pores (highly porous materials with a range of pore size) and interconnected structures with a morphology like ECM capable of supporting cell growth [39]. PVA has been chemically blended with HA and other natural polymers to improve its similarity with ECM. This results in interesting tailor-made materials [51,52,53]. Since HA has a pronounced hydrophilic character that causes fast degradation rates and weak mechanical properties, it has been suggested that HA must be used after chemical modification and/or crosslinked with organic or inorganic polymers [6,22]. Unfortunately, few studies have been conducted on electrospun pristine HA materials [47] since the high viscosity and surface tension at relatively low concentrations make it a polymer that is very difficult to electrospun [48]. 

**Figure 5:**
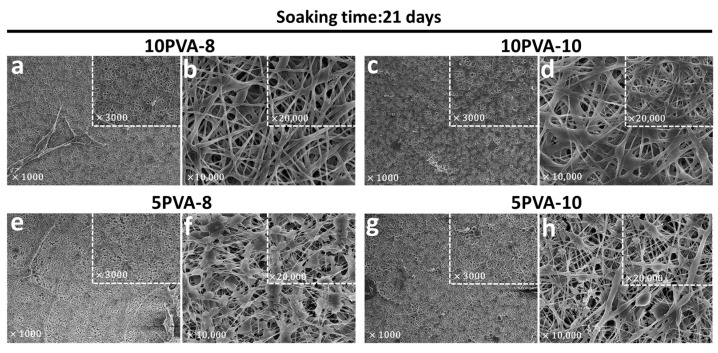
Electrospun PVA–HA–siloxane hybrid nanofibers morphology after 21 days of soaking into the culture medium. (**a**,**b**) Mats with hybrid nanofibers 10:1 PVA:HA and spinning distance of 8 cm. These mats show more resistance to degradation in the culture medium, and their morphology remained. (**c**,**d**) Mats with hybrid nanofibers 10:1 PVA:HA and spinning distance of 10 cm. The nanofibers of these mats lost their cylindrical shape after soaking. (**e**,**f**) Mats with hybrid nanofibers 5:1 PVA:HA and spinning distance of 8 cm. The beaded pattern of these nanofibers was lost and mats showed fractures after soaking. (**g**,**h**) Mats with hybrid nanofibers 5:1 PVA:HA and spinning distance of 10 cm. The fibers of these mats lost their shape and were thinner compared to mats before immersion. In all images, the small squares show a magnification of the same sample.

To prove that PVA–HA–siloxane hybrid nanofibers exhibit good resistance to degradation, Figure 5 displays SEM images of the PVA–HA–siloxane mats after 21 days of cell culture in supplemented α-Minimum Essential Medium Eagle, which is used for the growth of osteoblasts.

After 21 days of immersion, the PVA–HA–siloxane hybrid nanofiber mats maintained their morphological structure, as previously described in Figure 4. Most of the fibers in the mats with higher PVA content (10PVA-8 and 10PVA-10) maintain their small diameters. However, some of them are thicker and flatter; have lost their cylindrical shape and appear to blend into each other. Shape loss and blending seem to be higher in the 10PVA-10 mats. On the other hand, mats with lower PVA content (5PVA-8 and 5PVA-10) show fractures. In addition, their “ribbon-like” fibers are thinner compared to the fibers before immersion. This suggests hydrolytic degradation. The bead structures also experienced changes, and their elongated droplet shapes turned into several flat shapes and sizes. Hydrolytic degradation appears to be greater in the 10PVA-8 mats.

According to the observed changes, the variation in PVA:HA ratios affects the physicochemical characteristics such as swelling behavior, hydrolytic degradation, mechanical flexibility, and protein adsorption of the hybrid mats. Mats with lower HA content (10PVA-8, 10PVA-10) exhibited less hydrolytic degradation on their nanofibers due to (1) the PVA affinity with PDMS silicone which boosts their hydrophobic behavior and (2) that PVA contributes to the reduction of HA degradation rate, avoiding its rapid dissolution [54]. On the other hand, mats with higher HA content (5PVA-8, 5PVA-10) showed more hydrolytic degradation on their nanofibers since the PVA ratio decreased and HA concentration increased within the hybrid system, tailoring degradation rates in unhybridized HA macromolecules. Siloxane–inorganic crosslinking in all mats provides excellent physical and chemical support to allow cell growth.

### 3.3. Cell Viability on PVA–HA–Siloxane Nanofiber Mats

**Figure 6:**
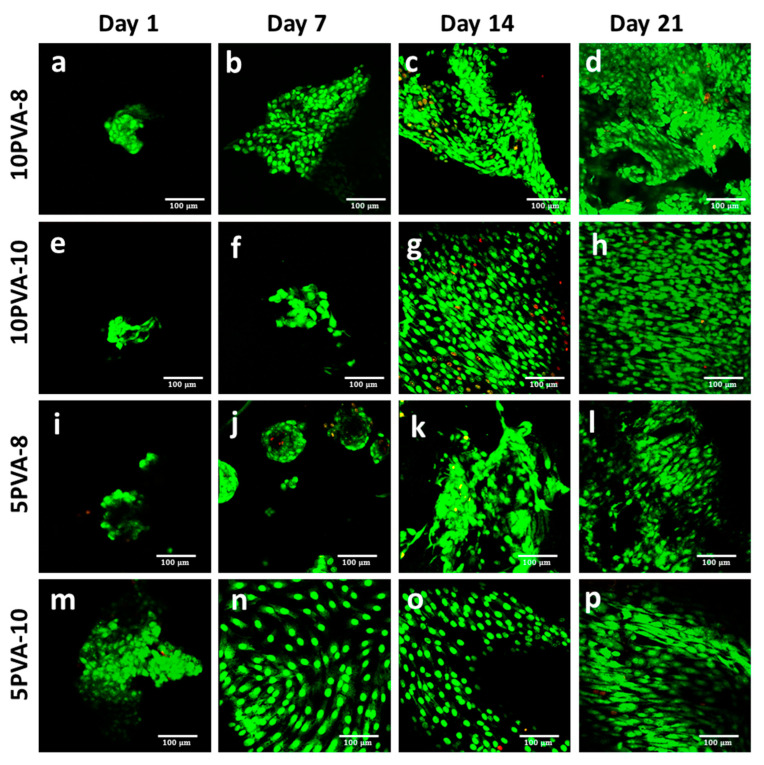
Viability of osteoblasts grown on PVA–HA–siloxane nanofiber mats. Osteoblasts were incubated on nanofiber mats for 1, 7, 14, and 21 days. After incubation, the cells were stained with SYTO9-PI solution. Live cells were dyed green, and dead cells were red. (**a**–**d**) Representative images of 10PVA-8 mats; the cell viability was maintained in these mats only on day 14, and some cells lost their viability. (**e**–**h**) Representative images of 10PVA-10 mats; cells on these mats conserved their viability until day 21, and a few cells lost their viability at day 14. (**i**–**l**) Representative images of 5PVA-8; cells on these mats maintained their viability until day 21, and at day 7, spheroid-like 3D structures were evident. (**m**–**p**) Representative images of 5PVA-10 mats; cell viability was maintained in these mats, and cells formed a circular growth pattern. In all samples, the increase in number of cells became evident over time. The images show the cells in a representative focal plane of the 3D nanofiber mats. The bar in all images represents 100 μm.

Biological responses to osteoblast cells, such as enhanced cell adhesion, cell growth stimulation, and calcium deposition, were studied in PVA–HA–siloxane hybrid nanofiber mats. The viability of osteoblast cells grown on nanofiber mats was evaluated by immunofluorescence using SYTO9 and PI. Confocal images showed viable cells for all fiber mats since the first day. However, the 5PVA-10 mats exhibited the maximum number of cells. On day 14, 10PVA-8 and 10PVA-10 mats showed a smaller number of dead cells. On day 21, all materials efficiently supported the viability and proliferation of osteoblasts. Decreasing the ratio of PVA in the PVA–HA–siloxane nanofiber mats had a positive effect for the first time on the adhesion and cell proliferation behavior since the HA ratio increased, boosting their biological properties. These results confirm the role of HA as a base component in scaffolds for tissue regeneration. Because this polymer is a natural component in ECM, it offers a similar extracellular environment to seeded cells. This results in cell adhesion, proliferation, and differentiation enhancement. Several studies have observed osteogenic-enhancing properties such as cell proliferation and differentiation in hyaluronic acid-based scaffolds [55,56]. These osteogenic properties of hyaluronic acid are molecular weight- and concentration-dependent [56]. Other studies have indicated that HA favors osteogenesis [57,58]. Having HA as part of the scaffold components can give the material the ability to bind water to it [59]. However, having chemically modified HA with APTES and then inorganically crosslinking it with siloxane molecules makes the HA less hydrophilic and more resistant to hydrolytic-degradation. The chemical interaction of organic and inorganic components gives the electrospun scaffolds the power of mechanical support and chemical stability to boost their intrinsic osteogenic properties (cell proliferation), as observed in Figure 6.

### 3.4. Cytoskeletal Arrangement of Cells Adhered onto PVA–HA–Siloxane Nanofiber Mats

**Figure 7:**
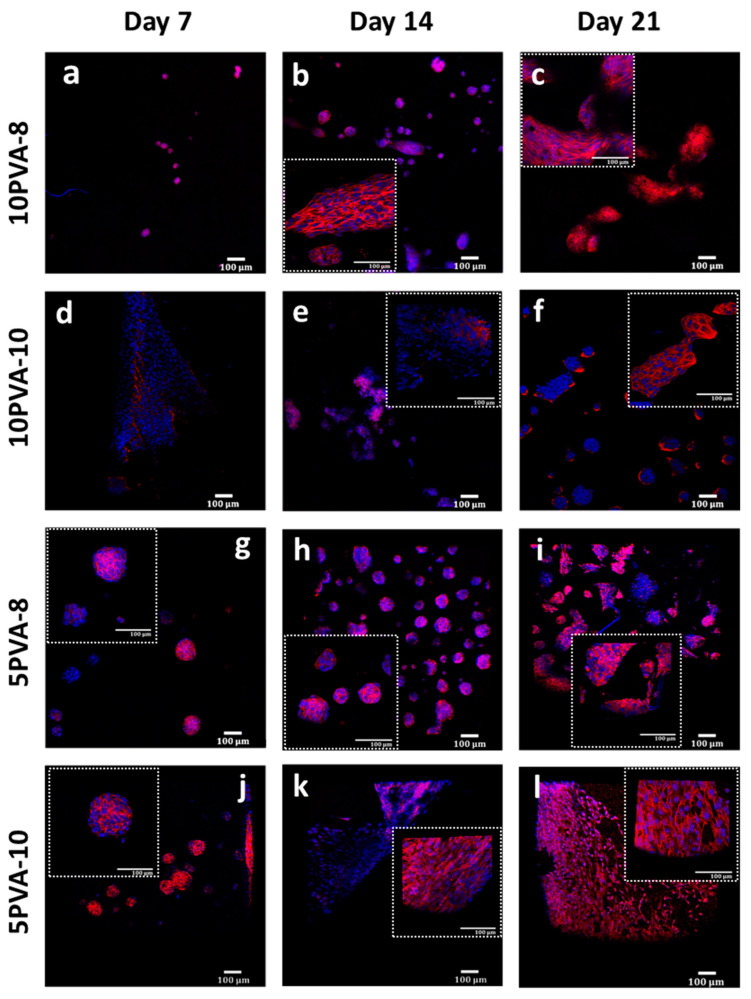
Actin cytoskeleton of osteoblasts grown on PVA–HA–siloxane nanofiber mats. (**a**–**c**) Representative images of cell growth on 10PVA-8 mats. The actin cytoskeleton was more evident at 14 and 21 days, and some spheroid structures were evident at 14 days. (**d**–**f**) Representative images of actin stain of the cells grown on 10PVA-10 mats. Actin filaments distribution was evident from 7 days and maintained until 21 days. (**g**–**i**) Images of cells grown on 5PVA-8 mats. The formation of spheroid-like 3D structures was evident for 7 days, and this pattern was conserved until 21 days. Actin filaments are present in these structures. (**j**–**l**) Representative images of osteoblasts grown on 5PVA-10 mats. The formation of 3D structures was visible for 7 days. The homogenous distribution of actin filaments was evident in cells at 14 and 21 days. Actin filaments were stained with rhodamine-phalloidin (red), and cell nuclei were labeled with DAPI (blue). The images represent only one focal plane of materials and represent two experiments.

The actin cytoskeleton is highly implicated in several cellular processes, such as cell survival, proliferation, and motility. These processes are related to the formation of tissues. According to this, the morphological analysis of MG-63 osteoblasts seeded on PVA–HA–siloxane biomaterials was evaluated by staining actin filaments. On the 7th day, cells developed an adherent monolayer on the 10PVA-10 mats. The actin filaments were organized as parallel bundles alongside cells, and they kept this pattern till day 21. Interestingly, on the rest of the materials (10PVA-8, 5PVA-8, and 5PVA-10) cells grew up with the shape of spheroid-like 3D structures, which have been appreciated since the 7th day. Cultured cells on 10PVA-8 and 5PVA-8 mats maintained this spheroid-like morphology until day 14, and 5PVA-8 mats until day 21. Finally, cells matured in all mats and they were found to be in close interaction on day 21. Their actin cytoskeleton was found to be well organized and to support the phenotypic morphology of osteoblasts (Figure 7).

The assembly of spheroid-like structures on HA-based composite materials has been previously reported [60]. These structures reproduce the 3D architecture of tumors, which is why their use as models for screening therapeutic drugs against cancer has been highlighted [60,61]. The cell proliferation and its successful growth on nanofibers depend on the establishment of the interaction of HA with the cellular surface marker CD44 receptor. CD44 is a transmembrane receptor expressed by many cells and binding ligands such as hyaluronic acid [62]. This interaction promotes the production of autocrine growth factors favoring cell proliferation [63]. It is also directly related to proteins that participate in the cytoskeleton rearrangement, such as Rac and Rho proteins [64]. Thus, CD44 interacts with the cytoskeleton, and confers motility to cells and further migration by (1) opening up tissue spaces through which cells move and (2) enabling cells to move [59]. The PVA–HA–siloxane electrospun scaffold is (1) the surface on which osteoblasts adhere and (2) the extracellular microenvironment. Having HA as a component in the PVA–HA–siloxane mats influences cell–cell and cell–scaffold interactions by enhancing cell motility and cell migration across the nanofiber mat. This results in granulation tissue formation [62]. Figure 7 shows the actin filaments of osteoblasts and how they are organized during the assay. It is observed that osteoblasts migrate and proliferate across the mats, but they settle down in the form of parallel cell-bundles; these are well organized and in close interaction to simulate granulation tissue formations. Mats with a higher HA ratio, especially the 5PVA-8 mat, showed more spheroid-like cell-bundles. This highlights the role of HA in the formation of these structures as part of the scaffold component.

### 3.5. Cell Attachment onto PVA–HA Hybrid Nanofiber Mats

**Figure 8:**
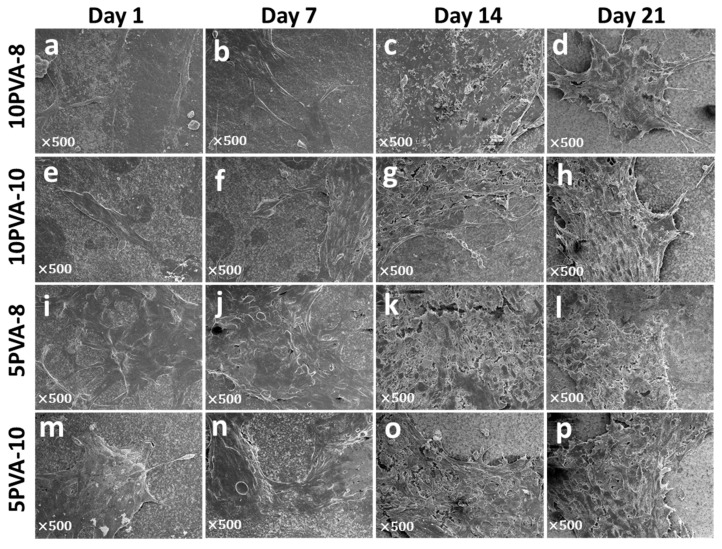
Ultrastructure of osteoblasts spread on PVA–HA–siloxane nanofiber mats. Osteoblasts attached to the surface of the fiber mats were observed using the conventional SEM technique. (**a**–**d**) Representative images of osteoblasts growth on 10PVA-8 mats; cells are visibly adhered on these mats. At the first days (1 to 7 days) few cells are found, and cellular density is more apparent at 14 and 21 days. Membranal filopodia are present at 21 days. (**e**–**h**) Ultrastructure morphology of cell growth on 10PVA-10 mats. On these mats, osteoblasts are more spread out, and cellular density is evident from 7 days. (**i**–**l**) Growth osteoblasts on 5PVA-8 mats; a greater cell density is evident in these mats with typical osteoblasts morphology. (**m**–**p**) Ultrastructure morphology of osteoblasts grown on 5PVA-10 mats; clusters of cells are visible on these mats, and cellular contact is more closed from cell to cell.

To confirm osteoblasts’ attachment onto nanofiber mats and observe their morphology, they were analyzed by scanning electron microscopy, as shown in Figure 8. In all mats, cells showed the typical spindle-shaped morphology of osteoblasts. In some cases, cells presented large projections such as filopodia, and they grew up covering the total surface of the nanofiber mats. It is notable that on the 5PVA-8 mat, cells proliferated and spread in the form of multilayers resembling 3D structures. The formation of multilayer-cell 3D structures suggests the beginning of tissue growth, supporting granulation tissue formation and confirming the outstanding biological properties of the PVA–HA–siloxane hybrid scaffolds.

Osteoblasts adhere to the electrospun mat scaffold, and become to be the extracellular microenvironment. Since HA is a natural ECM component, it provides a similar extracellular environment, helping osteoblasts to adhere and proliferate, as observed in Figure 8. The novel 3D organic–inorganic siloxane network crosslinking the sodium hyaluronate provided the polysaccharide chains with strong chemical bonds that restricted their natural hydrophilicity and gave stiffness to the nanofiber mats [6]; this supports cell attachment, cell proliferation, and growth of new tissue. Other studies have reported similar results in HA-based nanofiber mats. For example, the addition of hydroxyapatite and chitosan in electrospun PVA/HA nanofiber mats increases the WI38 cell adherence and proliferation, and this supports its properties for bone regeneration [65]. In addition, Pangon and colleagues reported the functionality of chitin whiskers to enhance the mechanical properties of chitosan/PVA nanofibers and improve osteoblasts´ viability and proliferation [66]. In another PVA-based scaffold, cell adhesion was improved by adding HA, and the increase in cell proliferation was due to the enrichment of hydroxyapatite [67]. Interestingly, the addition of hydroxyapatite was not required for cell adhesion and proliferation of osteoblasts in mats fabricated in this work. Moreover, in electrospun HA-based nanofiber with the incorporation of glycosaminoglycans (GAGs), cells such as human keratinocytes, fibroblasts, and mesenchymal stem cells firmly adhered to the nanofiber showed high cell viability and cellular proliferation [68]. 

### 3.6. Differentiation of Osteoblasts Grown on PVA–HA Hybrid Nanofiber Mats

**Figure 9:**
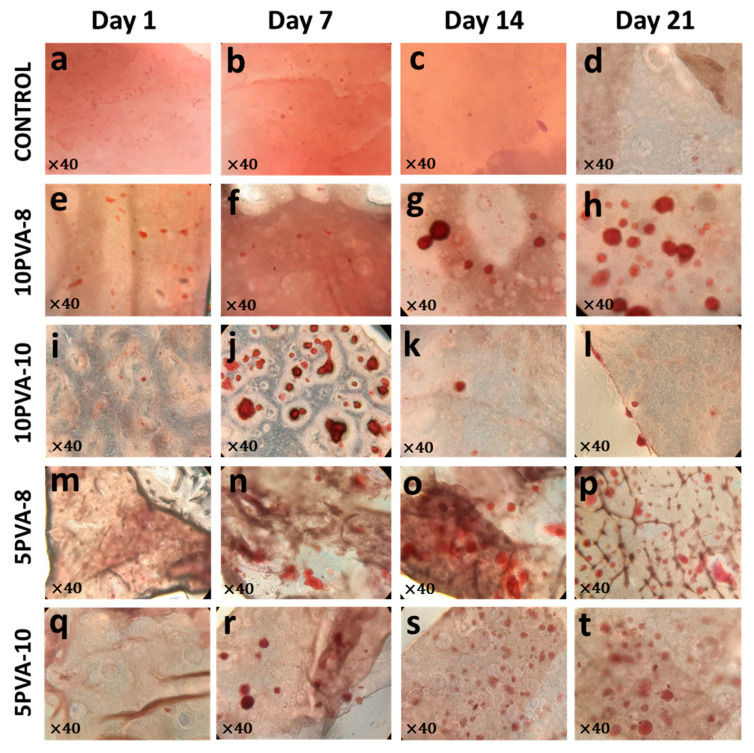
Calcium deposits produced from osteoblasts grown on PVA–HA–siloxane hybrid nanofiber mats. Calcium deposits were stained with the alizarin red solution. (**a**–**d**) Representative images of mats without cells; no alizarin red stain was visible on control mats. (**e**–**h**) Representative images of calcium deposit formation on 10PVA-8 mats. The mineralization increased over time; on days 14 and 21, the calcium deposits were more evident. (**i**–**l**) Formation of calcium deposits on 10PVA-10 mats; remarked at 7 days on. (**m**–**p**) Mineralization on 5PVA-8 mats was evidenced by alizarin stain, the calcium deposits were more apparent from 7 days to 21 days. (**q**–**t**) Mineralization induced on 5PVA-10 mats; the increase of alizarin stain was prominent from 7 until 21 days.

During cell maturation, osteoblasts differentially expressed several proteins. Some of these proteins induce mineralization in the extracellular matrix, and calcium deposits are accumulated to promote the formation of hydroxyapatite crystals. To evaluate the effect of PVA–HA hybrid nanofiber mats on the adhered osteoblasts´ ability to reach a mature stage, mineralization nodules were evaluated by Alizarin-staining, as shown in Figure 9. Alizarin-stained calcium deposits were found in all nanofiber mats. Their initial formation was more evident on the seventh day, and their abundant presence remained until the 21st day, except for the 10PVA-10 mat, in which fewer mineralization nodules were found on days 14 and 21. The favorable mineralization in HA-based nanofibers has been previously reported, for example, in poly(l-lactic acid)-co-poly(ε-caprolactone)-silk fibroin-hydroxyapatite-hyaluronic acid (PLACL–HaP–SF–HA) scaffolds, the osteoblastic bone formation was attributed to hyaluronic acid and the increase of mineralization was due to incorporation of silk fibroin on days 10 and 15 [69]. The role of HA in supporting the differentiation and calcium mineralization has also been highlighted in HA + FmocFRGD/PCL electrospun fibers [70]. The calcium deposits resulting from osteoblast maturation and differentiation inducing mineralization in the ECM confirm the outstanding biological properties of the PVA–HA–siloxane hybrid mats. HA working as a fundamental component within the nanofiber mats enhances the osteogenic effects of the tested hybrid scaffolds. It can be observed that HA acts as the vector for osteoinductive properties in the hybrid membranes.

## 4. Conclusions

PVA–HA–siloxane hybrid nanofiber mats with outstanding biological properties were successfully synthesized. The improved biological properties were enhanced by the inorganic–siloxane chemical crosslinking of PVA–HA polymeric chains; this prevented their hydrolytic degradation and metabolism, provided support to cells, and boosted HA intrinsic osteogenic properties. The PVA, as a synthetic polymer with good biological properties, helped the electrospinning of HA. Hybrid nanofiber mats with a higher PVA ratio (10PVA-8 and 10PVA-10) show less cell growth during the seven days of culture but as time passes, cell growth significantly increases. On the other hand, hybrid nanofiber mats with lower PVA ratio (5PVA-8 and 5PVA-10) show higher cell growth from the first day of culture. However, these mats also presented a higher degradation at the end of the assay, which is attributed to the higher HA ratio within the fiber mats. All the hybrid mats support cell adhesion, cell proliferation, and cell differentiation (cell maturation and calcium deposits) during the 21 days of the assay. It is noteworthy to state two relevant facts: (1) the 5PVA-8 mat shows 3D spherical osteoblast morphology, suggesting 3D tissue growth formation, and (2) the functionality of the cells grown on the nanofiber mats was independent of the addition of both factors and stimulating-cell-growth cytokines, making these materials excellent candidates for their use in bone tissue repair.

## Data Availability

The data presented in this study are available on request from the corresponding authors.
